# PlethAugment: GAN-Based PPG Augmentation for Medical Diagnosis in Low-Resource Settings

**DOI:** 10.1109/JBHI.2020.2979608

**Published:** 2020-11-04

**Authors:** Dani Kiyasseh, Girmaw Abebe Tadesse, Le Nguyen Thanh Nhan, Le Van Tan, Louise Thwaites, Tingting Zhu, David Clifton

**Affiliations:** Engineering Science Department, University of Oxford, Oxford OX3 7DQ, U.K.; Oxford University Clinical Research Unit, Ho Chi Minh City 700000, Vietnam; Engineering Science Department, University of Oxford, Oxford OX3 7DQ, U.K.

**Keywords:** Conditional generative adversarial networks, data-augmentation, time-series, photople-thysmogram, low-resource

## Abstract

The paucity of physiological time-series data collected from low-resource clinical settings limits the capabilities of modern machine learning algorithms in achieving high performance. Such performance is further hindered by class imbalance; datasets where a diagnosis is much more common than others. To overcome these two issues at low-cost while preserving privacy, data augmentation methods can be employed. In the time domain, the traditional method of time-warping could alter the underlying data distribution with detrimental consequences. This is prominent when dealing with physiological conditions that influence the frequency components of data. In this paper, we propose PlethAugment; three different conditional generative adversarial networks (CGANs) with an adapted diversity term for the generation of pathological photoplethysmogram (PPG) signals in order to boost medical classification performance. To evaluate and compare the GANs, we introduce a novel *metric-agnostic* method; the *synthetic generalization curve*. We validate this approach on two proprietary and two public datasets representing a diverse set of medical conditions. Compared to training on non-augmented class-balanced datasets, training on augmented datasets leads to an improvement of the AUROC by up to 29% when using cross validation. This illustrates the potential of the proposed CGANs to significantly improve classification performance.

## I Introduction

AUCITY of data and class imbalance drastically hinder the performance of modern machine learning algorithms [1], [2]. In the medical domain, the relatively low number of patients enrolled in experimental trials, among other reasons, limits the amount of data collected. This is even more pronounced in low-resource clinical settings where high financial and infrastructural constraints exist. To overcome this obstacle, the use of wearable sensors capable of continuous monitoring of physiological signals such as the photoplethysmogram (PPG) has experienced a rise [3]. The amount of data limits researchers from capitalizing on deep learning approaches which are known to be data-hungry [4] and which have produced promising results in cognate disciplines. Therefore, generating class-specific medical time-series data may help in alleviating some of the aforementioned obstacles.

Data augmentation, the process of generating new data from the existing data is common in computer vision [5] where images are flipped and rotated at various angles in order to augment the dataset and act as a form of regularization. Given its positive impact on classification performance [6], it has been used for various tasks involving deep learning for medical images such as segmentation [7] and liver lesion classification [8]. In the time-domain, on the other hand, the addition of noise and time-warping is performed [9]. Such approaches can lead to unwanted changes in the physiological signals, changing the underlying data distribution in a manner that might affect subsequent classification. This is especially problematic when dealing with medical conditions such as hand-foot-mouth (HFM) disease and tetanus (both of which are especially prevalent in low-resource settings) that impact the nervous system, and consequently, the frequency components of the physiological signal. Consequently, a generative process that accurately and *realistically* represents the data is needed.

Recently, generative adversarial networks (GANs) have been used for data augmentation purposes [10] given their ability to capture the underlying data distribution. Conditional GANs (CGANs) for data augmentation, however, have not been fully explored, let alone in the medical domain.

### Contribution

**Fig. 1:**
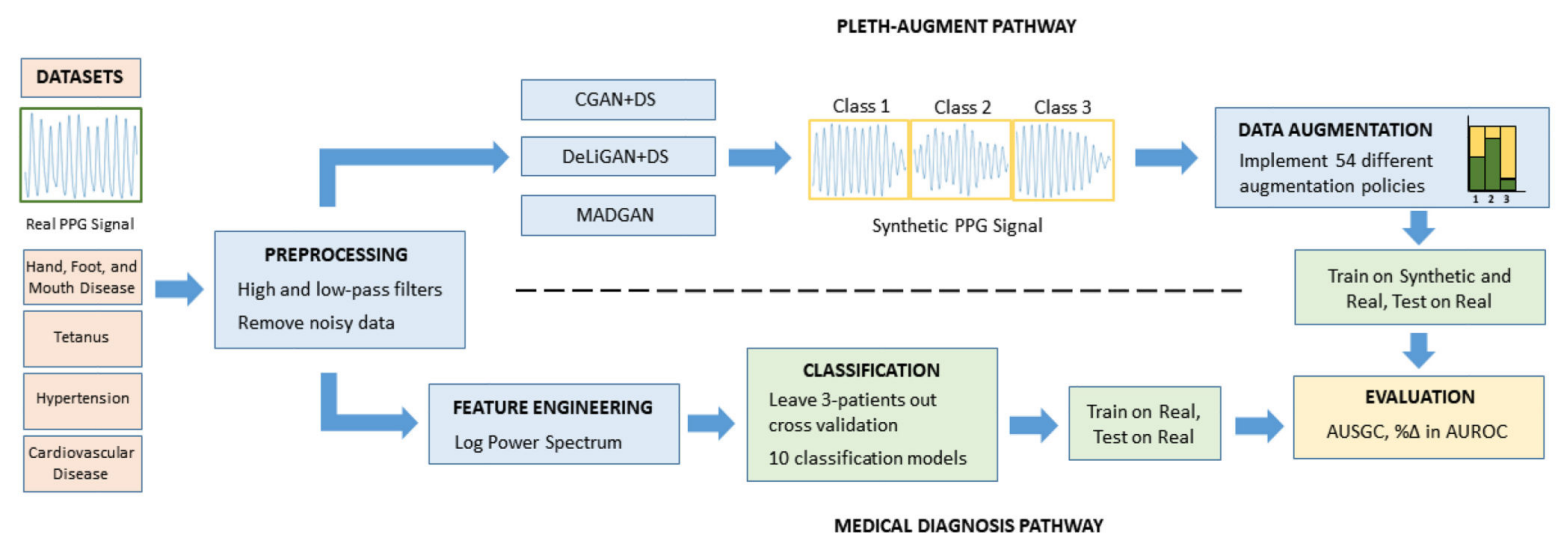
Illustration of Pipeline. Synthetic data generated by the three different CGAN models are used to augment the original dataset for a 3-way classification problem.

In this paper, we follow the pipeline in Fig. 1 by proposing several CGANs inspired by work in other fields [11]–[13] and adapt them to generate disease-severity-specific photoplethysmogram signals. We use the resulting synthetic signals to augment a dataset and improve upon the baseline performance. Finally, we introduce the *synthetic generalization curve*, a novel and generalizable method for evaluating and comparing the performance of GANs.

## II Related Work

GANs [14] were first introduced as a generative model based on a minimax formulation where two networks, the generator and discriminator, engage adversarially to outsmart one another. Shortly after, CGANs [11] were introduced as simple extensions to GANs where the generated data is conditioned on a certain variable such as a class, time-stamp, etc.

### A Conditional Generative Adversarial Networks (CGANs) for Time-Series

GANs have been successful in generating medical *images* for the purpose of augmenting datasets [15]. A recent review by Yi *et al*. [16] summarizes the state-of-the-art in that domain. Given that medical image synthesis is beyond the scope of this paper, we solely focus on applications to time-series data. Although in its infancy, the application of CGANs for time-series data has seen a recent rise in activity. CGANs have been used to generate weather data conditioned on specific scenarios [17] and to generate wind and solar energy production over time conditioned on environmental variables [18]. Others introduce MuseGAN [19] to generate track-specific polyphonic music. Although their task is temporal, their data representations lack high sampling rates usually experienced in physiological signals. Others [20] attempt to model the potential trajectories of humans over time using an LSTM-based generator and discriminator. In the medical domain, the work in [21] uses a 1D convolution-based GAN to generate electroencephalogram (EEG) brain signals. Inspired by this work, others generate synthetic epileptic brain activity signals [22] and EEG signals [23] specifically to improve classification models. Others [24] use a GAN to generate an open-source AND privacy-protected vital sign dataset. In [25], PPG and electrocardiogram (ECG) data are generated using the 2D convolution-based DCGAN. Here, time-series data are converted to images before being input into the GAN. Both of these works, however, do not aim to generate class-specific signals. Although the authors in [26] use a conditional DCGAN to generate EEG data, they perform their operations in the imaging domain and do not evaluate the representativeness of the synthetic EEG data. Closest to our work is that of [27] which uses an LSTM-based CGAN to produce various time-series data, including sine waves, some medical data, and sequential MNIST benchmark data. The medical time-series generated, however, is of summary numerics such as heart rate and oxygen saturation as opposed to high frequency medical data. Notably, they introduce an evaluation metric known as “Train on Synthetic, Test on Real” (TSTR) which we build upon in our work. Lastly, although not used for time-series, DSGAN [28] involves a diversity sensitivity term that rewards conditional GANs for diverse data generation.

### B Data Augmentation for Time-Series

Given the improved results associated with data augmentation in computer vision [29], recent work converts time-series into image-representations [30], [31]. The work in [9] provides a good overview of data augmentation methods to employ on time-series data from wearable sensors. This includes random jitter, window-slicing, changing permutations, and time-warping. The latter is used before implementing a convolutional neural network [32] and to boost the performance of a deep ResNet classification network [33]. Unfortunately, the aforementioned approaches could be detrimental in our application especially when dealing with physiological conditions that impact a signal’s frequency component. The addition of noise from a Gaussian distribution with varying standard deviations has been used to improve the classification performance of three different models (SVM, LeNet, ResNet) on various datasets [18]. While promising, the results are inconsistent and the methdology does not seem to generalize well. In the music domain, authors in [34]–[36] leverage an audio degradation toolbox that introduces perturbations to the original data. To avoid domain-specific augmentation problems, additive noise is proposed [37], in addition to interpolation and extrapolation in the feature space as a form of data augmentation before data are fed into a classifier. In contrast to traditional augmentation approaches, an end-to-end model that learns invariant transformations to apply to the original data is proposed [38]. Although this resulted in minor classification improvement, their approach was limited to low-frequency data (1 sample per hour).

## III Experimental Methods

We are focused on a conditional GAN-based time-series data augmentation methodology in an effort to improve the performance of classification models. To achieve this purpose, we have chosen and adapted three various conditional GAN models that have had success in generating diverse images. When training such models, we leveraged advice pertaining to improving training stability and performance [39].

### A Encouraging Intraclass Diversity

The importance of generating sufficiently diverse class-specific data motivated us to adapt a reward term introduced in [28] by making it class-specific. Classes can be defined arbitrarily and may depend on the dataset used. In the context of this paper, for instance, the classes represent various disease severity levels.

(1) $${ℒ}_{DS}=-E_{c}[E_{z_{1},z_{2}}[\frac{\left||G(z_{2}|c)-G(z_{1}|c)|\right|}{z_{2}-z_{1}}]]$$

where the outer expectation is with respect to all classes, *G* represents the generator network, and *z*_1_ and *z*_2_ represent any two input noise vectors belonging to the same class *c*. Intuitively, this term rewards the generator according to how sensitive it is to a change in input. Extreme mode-collapse, for instance, results in a sensitivity of zero because the same output would be generated for two different noise inputs (*G*(*z*_2_|c*c*) = *G*(*z*_1_|*c*)). Thus a null reward value is returned. We incorporate this term into our proposed CGAN models in the hope of encouraging intraclass diversity.

### B Encouraging Interclass Diversity

#### 1) Vanilla CGAN With Diversity Sensitivity

The “vanilla CGAN” incorporates the conditional variable at any point within the generator *G* and/or discriminator *D* network. We opted to concatenate a one-hot encoding of the class of the PPG to the input of the generator. Our generator was trained using a loss function that consists of three terms; i) a Jensen-Shannon loss ℒ_*JS*_ that penalizes the network for generating unrealistic synthetic data $\hat{x}$, ii) an auxiliary cross-entropy loss that penalizes the network for generating data that cannot be correctly classified as the ground truth *k*, and iii) our proposed class-specific diversity sensitivity loss (1) that penalizes the network for not generating synthetic data that is diverse.

(2) $${ℒ}_{G}={ℒ}_{JS}-E_{\hat{x}\sim P_{g}}[\log(p(y=k|\hat{x}))]+\lambda_{div}{ℒ}_{DS}$$

(3) $${ℒ}_{JS}=-E_{\hat{x}\sim P_{g}}[\log(D(\hat{x}))]$$

where *P*_*g*_ represents the distribution of synthetic data and *λ*_*div*_ is a hyperparameter that determines the degree of diversity sensitivity. Independently of the generator, the discriminator was trained using a loss function that also consists of three terms; i) a Wasserstein loss [40] that penalizes the network for classifying the synthetic data as realistic and the real data as synthetic, ii) a gradient penalty of zero [41] that was found to improve training stability, and iii) an auxiliary cross-entropy loss that penalizes the network for incorrectly classifying the real data.

(4) $$\begin{array}{r} {ℒ}_{D}=E_{\hat{x}\sim P_{g}}[D(\hat{x})]-E_{x\sim P_{r}}[D(x)]+E_{\bar{x}}[||\nabla_{\bar{x}}D(\bar{x})|{|}^{2}]\\ -E_{x\sim P_{r}}[\log(p(y=k|x))] \end{array}$$

where *P*_*r*_ represents the distribution of the real data, ∇ represents the gradient operator and $\bar{x}=\alpha x+(1-\alpha )\hat{x}$ is a linear combination of the real and synthetic data with *α* ~ *U* (0, 1), as suggested by the original authors.

#### 2) DeLiGAN With Diversity Sensitivity

DeLiGAN [12] is proposed to deal with diverse and limited data regimes. As part of the generative model, the parameters of a Gaussian Mixture Model are learned through training. We remove the variance regularization term originally introduced and replace it with the diversity sensitivity term mentioned earlier. Furthermore, we revert to the traditional Jensen-Shannon loss term. Our generator and discriminator loss are represented by (2) and (4), respectively.

#### 3) MADGAN

MADGAN [13] is proposed as a way to explicitly generate data from different classes. In order to do this, as many generators as there are classes are introduced. Our generator loss consists of a Jensen-Shannon loss term of the form in (3) for each generator and is as follows:

(5) $${ℒ}_{G}={ℒ}_{JS_{1}}+{ℒ}_{JS_{2}}+{ℒ}_{JS_{3}}$$

The discriminator is tasked with identifying whether the data is real or synthetic, and if it is the latter, to further identify the generator from which it came. Our discriminator loss is the same as that suggested in the original paper.

## VI Evaluation Methods

There are many ways to evaluate GANs as summarized in [42]. Although we take inspiration from some of these techniques, our focus does not lie here. Given our desire to quantify the potential improvement in medical diagnosis offered by data augmentation, we build upon the work introduced in [27] and further propose a novel evaluation method.

### A GAN-Specific Evaluation

With time-series, in contrast to computer vision, assessing the quality and representativeness of synthetic data is not straight-forward. Moreover, a common pitfall of such networks is mode collapse where the generator fails to produce diverse samples; i.e., there exists a many-to-one or many-to-few mapping of random variable *z* to synthetic image $\hat{x}$. This is especially problematic in the conditional GAN case where some diversity is expected in the generated data. Thus, we evaluate our GANs by measuring the following:

#### 1) Representativeness of Synthetic Data

We use the kernel maximum mean discrepancy (MMD) [43], a common evaluation method for GANs that compares the similarity of synthetic data and real data. This similarity is quantified using a kernel function *K*, and in our case, we use the exponentiated quadratic.

(6) $$K(x,x\prime )=e^{-||x-x\prime |{|}^{2}}$$

where *x* and *x*′ are two vectors to be compared. If they are exactly the same, then the kernel function evaluates to one. The more dissimilar they are from one another, the smaller the value is, which is lower-bounded by zero. Since the original MMD metric fails to illustrate the more granular class-specific similarities, we introduce *M M D*_*c*_; a conditional MMD metric that allows us to compare class-specific performance across different GANs as shown below

(7) $$M\;M\;D_{c}=\underset{i\neq i\prime }{\sum }K_{ii\prime }-2\underset{i\neq j}{\sum }K_{ij}+\underset{j\neq j\prime }{\sum }K_{jj\prime }$$

where *K* is a kernel function that measures the similarity between its inputs, $K_{ii\prime }=K\left({\hat{x}}_{i}^{c},{\hat{x}}_{i\prime }^{c}\right),K_{ij}=\left({\hat{x}}_{i}^{c},x_{j}^{c}\right)$, *c* is a particular class, and $\hat{x}$ and *x* represent the synthetic and original data, respectively.

#### 2) Class Diversity

Variation in the generated data within and across the classes is important to detect, where the latter helps evaluate the *conditional* component of the CGAN. Since the MMD obscures this calculation, we explicitly calculate it through exponentiated quadratic kernels.

### B) Train on Synthetic and Real, Test on Real

We call the process of training on a dataset augmented with synthetic data and testing on the real dataset “Train on Synthetic and Real, Test on Real” (TSRTR). The outcome of this, when compared to a baseline, “Train on Real and Test on Real” (TRTR), allows us to see the effect of the data augmentation policy, which could be negative as observed in [44]. We define a data augmentation policy as a set of three parameters that dictate how to augment the original data: i) choice of class to imbalance, ii) degree of synthetic imbalance, and iii) ratio of synthetic to real data. The complete list of policies can be found in Section-VII of the Supplementary Material. Such an evaluation is performed using leave N-patients-out cross validation on 10 diverse classification models; Naive Bayes, Linear and Quadratic Discriminant Analysis, k-Nearest Neighbours, Logistic Regression, Support Vector Machines, Decision Tree, Random Forest, Adaboost, and Multilayer Perceptron. Mathematically, for a certain augmentation policy and for all classification models *M*, we calculate the percent change in a metric of interest.

(8) $${\text{\%Δ}}_{M}=\frac{X_{TSRTR}-X_{TRTR}}{X_{TRTR}}\cdot 100$$

where *X* can be any desired metric such as AUROC, and *X*_*TSRTR*_ and *X*_*T* *RTR*_ represent the metric value on a validation set when *training* on an augmented dataset and a non-augmented dataset, respectively.

### C Synthetic Generalization Curve

The above evaluation method is limited and simply provides us with the performance of an individual classification model for a particular augmentation policy. To obtain a holistic evaluation of all classification models for all augmentation policies, and thus provide a more realistic evaluation of any GAN, we propose the *Synthetic Generalization Curve*. Such a metric quantifies the extent to which all classification models *M* are over-or underperforming relative to a baseline. Mathematically, a point on the curve, which we call the synthetic generalization (*SG*), can be calculated as follows:

(9) $$\forall \varepsilon \;SG(X,\varepsilon )=\frac{1}{M}\overset{M}{\underset{m=1}{\sum }}\delta \left(X_{TSRTR}\geq (1-\varepsilon )X_{TRTR}\right)$$

where *δ* is the Kronecker delta function which evaluates to one if its argument is true and zero otherwise. The *SG* is performed for a particular augmentation policy *p*_*i*_ from the pool of policies *P*, and *ε* ≠ 1 dictates the comparison of the classification model in the augmented scheme *X*_*TSRTR*_ to the baseline (1 − *ε*)*X*_*T* *RTR*_ and varies according to user needs. For instance, when *ε <* 0, the SG represents the percentage of classification models in the augmented scheme that outperform those in the baseline by at least −*ε* · 100 percentage points. From this curve, a novel metric naturally follows: the *Area Under the Synthetic Generalization Curve* or *AUSGC*. This curve can be averaged over many augmentation polices to allow for a more realistic comparison of the performance of different types of GANs.

## V Experimental Setup

### A Dataset Description

#### 1) PPG From Patients in Vietnam With Hand-Foot-Mouth Disease

The PPG data were collected using a pulse oximeter (SmartCare Analytics Ltd., Oxford, UK) placed on the major toe of HFM-afflicted children between the ages of 3 and 6. Such data, sampled at a rate of 100 Hz, were collected from 74 patients upon admission to the pediatric intesive care unit, 6 hours after admission, and one day before discharge. Each data collection period was approximately 10 minutes in duration. Typically, HFMD severity is diagnosed based on medical criteria [45], [46]. For this dataset, diagnoses are performed by ICU physicians independently of the PPG waveform and consist of 3 classes in total.

#### 2) PPG From Patients in Vietnam With Tetanus

The PPG data were collected using a pulse oximeter (SmartCare Analytics Ltd., Oxford, UK) placed on the index finger of tetanus-afflicted adults. Such data were collected from 19 patients upon admission to the intesive care unit and one day before discharge. We only use the data from the first day of ICU admission. Each data collection period was approximately 24 hours in duration. Typically, tetanus severity is diagnosed based on clinical features outlined in the Ablett score [47]. For this dataset, diagnoses are performed by ICU physicians independently of the PPG waveform and consist of 3 classes in total.

#### 3) PPG From Patients in China With Cardiovascular Disease

The PPG data introduced in [48] were collected via a sensor used on CVD-afflicted patients between the ages of 21 and 86 and may be accessed in [49]. Such data, sampled at a rate of 1KHz, are collected from 219 patients in a clinical environment. Each patient has three data collection periods each of which is 2.1 s in duration. The 4-class diagnosis of hypertension includes; normotension, prehypertension, stage I, and stage II hypertension. In order to better compare results across datasets and architectures, we keep the number of classes consistent at 3 by combining the more similar data labelled normotension and prehypertension together. We did this to keep the network architecture consistent across datasets and because the aforementioned two classes are on the lower end of the severity of the medical condition.

#### 4) PPG From Physionet 2015 Challenge

The PPG data were the training data offered by the Physionet Challenge 2015 [50]. It consisted of recordings from 750 patients that suffered either of the following cardiac conditions; asystole, extreme bradycardia, extreme tachycardia, ventricular tachycardia, and ventricular flutter. The data, originally resampled to 250 Hz by the organizers of the challenge, were downsampled to 100 Hz for consistency with our other datasets. In effort to enable a fair comparison across datasets, we ensure that all tasks are a 3-way classification. Therefore, even though 5 cardiac classes exist, we choose to only distinguish between asystole, extreme bradycardia, and ventricular flutter.

### B PPG Data Representation

Medical conditions that are associated with autonomic nervous system dysfunction and heart rate variability can manifest themselves in the photoplethysmographic wave. A task force set up in 1996 [51] decided that five minutes of ECG data would be sufficient for a physician to discern such medical conditions. However, due to the lack of sufficient data and to avoid the curse of dimensionality [52], a shorter window was chosen to allow for an increased number of frames. Consequently, the PPG time-series data in this work is split into frames of *t* = 10 second duration. Given a sampling rate of *F*_*s*_, the length of each frame in samples becomes *F*_*s*_ *· t*.

### C CGAN Model Data

The discriminator of each CGAN model was fed a PPG frame of length *F*_*s*_ *· t* where *F*_*s*_ is 100 Hz and *t* is 5 seconds. These 500-dimensional frames were then reshaped according to the packing degree *p* used. Packing the frames consists of simply concatenating several frames along the time dimension and has been previously shown to improve the discriminator’s performance [53]. We found that a packing degree of 3 helped produce visually-realistic PPG data. A subset (20%) of the PPG frames from each dataset was used for training the CGANs.

### D Classification Models Data

PPG data were split into 5-second frames with 50% overlap. In other words, each frame was of length 500 and overlapped with the latter 250 points from the previous frame. Guided by the importance of the frequency components of the PPG signal, we used as input to the classification models the log of the one-sided power spectrum of the PPG frames. Therefore, the length of each input becomes *F*_*s*_/2, which in our case was 50.

### E Cross-Validation

For the evaluation of the classification models, we perform leave-3-patients-out cross-validation. Even though frames were split into 5-second segments and treated as independent from one another, the training and test folds were always split according to patients. This avoids patient-related data leakage. Moreover, each test fold consisted of PPG frames from exactly one patient from each of the three classes. Consequently, the total number of folds was equivalent to the lowest number of patients belonging to each class. In Table I, we outline the specific input data sizes *N* x*D* for each of the medical diagnosis tasks, where *N* and *D* represent the number of frames and dimensionality of the data, respectively. We also illustrate the degree of imbalance in the class labels.

### F Proposed CGAN Models Specification

We primarily use fully-connected layers for the generator and discriminator for all of the CGAN models implemented. The input to the generator is a 50-dimensional noise vector sampled from a standard Gaussian distribution. To reduce the noise present in the synthetic data, we add a 1D convolutional layer which acts as a low-pass filter before the final output of the generator, as described in [54]. We ultimately generate PPG signals with 500 time-steps. The specific network architecture can be found in Table II.

To make the discriminator more robust, we reshape batch outputs of the generator according to the packing degree *p* described in [53]. The DeLiGAN network consists of a random variable with three Gaussian mixture model components, one for each class. After experimentation, we found that when the mean vectors and covariance matrices were initialized randomly and isotropically (*σ* = 0.3), respectively, training was stabilized. Lastly, the MADGAN architecture has three generators with the same structure as that in Table II. We note the presence of two heads at the end of the discriminator; one for determining whether the data sample is fake or real, and another for predicting the appropriate class. Lastly, we choose *λ*_*div*_ = 1*e* − 6 as that appeared to stabilize training. By varying *λ*_*div*_, we briefly illustrate its effect on the GAN evaluation metrics in Section-I of the Supplemetary Material.

**Fig. 2:**
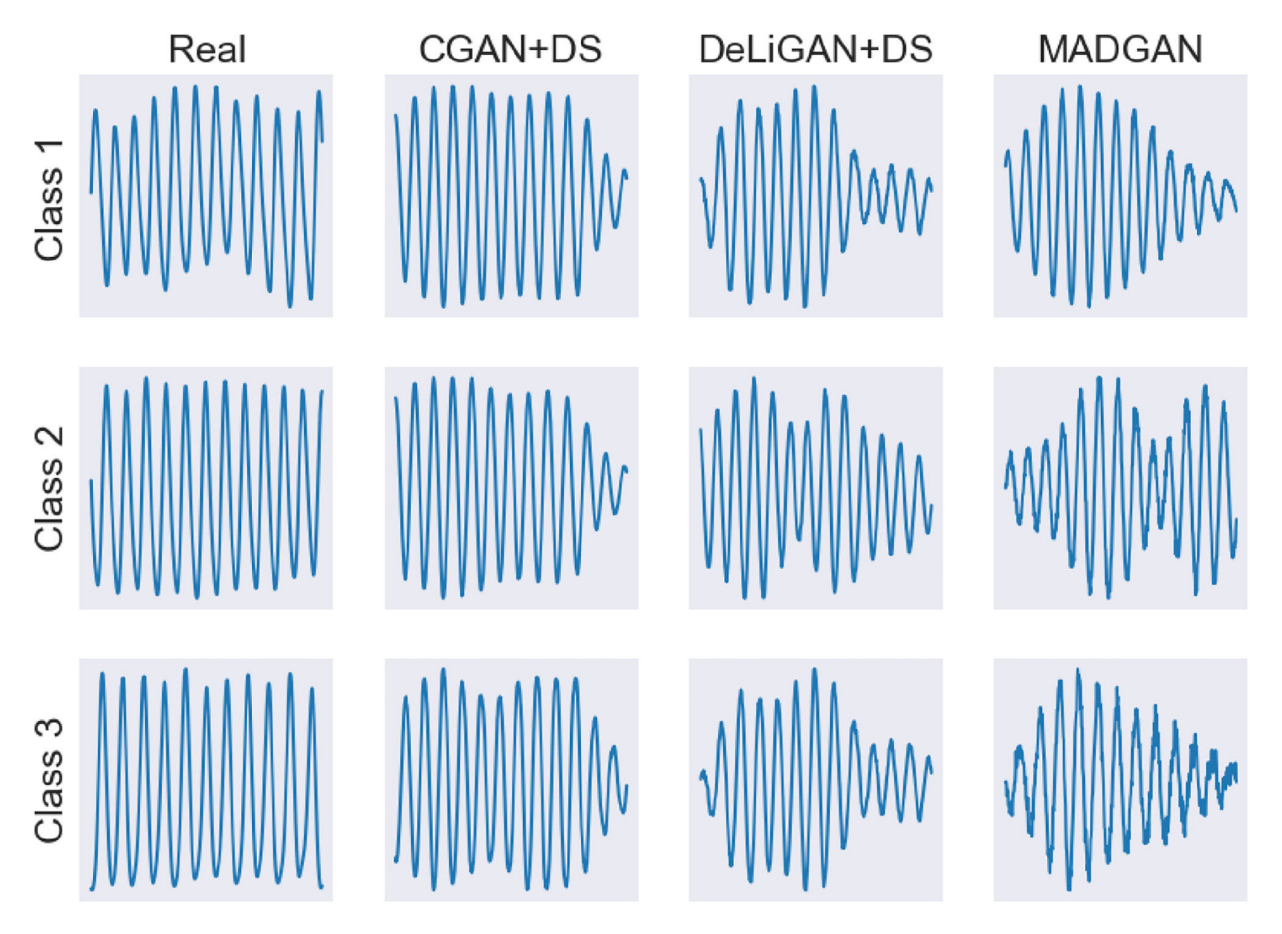
Randomly sampled class-specific real (HFM) and synthetic PPG data generated by each of the CGAN models. Samples are 5 s in duration. Note the ability of the CGANs to capture respiratory sinus arrythmia-induced amplitude modulation.

Sample outputs from each CGAN model are shown in Fig. 2 for the HFM dataset. Synthetic data for the remaining datasets can be found in Section-II of the Supplementary Material. In addition to the similarity in shape between the real and synthetic data, we draw the reader’s attention to a more subtle characteristic: amplitude modulation. Such low frequency changes in the PPG amplitude are hypothesized to represent respiratory sinus arrythmia [55], a naturally-experienced physiologial phenomenon. In some cases, our CGANs are able to capture this behaviour.

### G Baselines

The evaluation methods discussed earlier require a comparison to a baseline. Below is a description of the various baselines used. In all cases, the same training used for TSRTR is used for TRTR.

#### 1) Class Imbalanced Original Data

We employ TRTR while maintaining the original imbalance present in the dataset.

#### 2) Class Balanced Original Data

We employ TRTR while *balancing* the original imbalanced dataset. The balancing procedure is done by removing extra frames from the overpopulated classes. Motivation for this arises from improved performance due to a balanced dataset. Therefore, we report our augmentation results relative to this stronger baseline.

#### 3) SpecAugment

We implement the technique in [31] which focuses on the augmentation of time-series by masking randomly-chosen time and/or frequency bands in a spectrogram representation. Whereas the original work stops here, we then convert the spectrograms back into the time-domain using an inverse short time Fourier transform.

### H Effect of Data Augmentation - Hypotheses

In effort to find the ideal augmentation policy and whether that generalized across CGAN models and/or datasets, we formulated four different hypotheses. The sample size and normality of the data (supported via a Shapiro test) associated with such hypotheses motivated our use of the statistical t-test and ANOVA. Nevertheless, for extra precaution, their corresponding *non-parametric* statistical tests (Wilcoxon and Kruskal-Wallis) were also performed.

#### 1) CGAN Models

Without any prior knowledge, and given that the current CGANs have not been implemented on time-series data, there is no reason to believe one model should outperform the other. Therefore, we hypothesize that the results of the CGAN models will be similar.

#### 2) Training Set Imbalance

Given the work in [1], class imbalance is shown to degrade classifier performance. Therefore, we hypothesize that balanced training sets will outperform their unbalanced counterparts.

#### 3) Ratio of Synthetic to Real Data

Deep learning models are notorious for being data-hungry. Authors in [56], [57] illustrate the importance of training set size on non-parametric and deep learning models, respectively. However, a significant addition of synthetic data may result in a plateau [8] or even a worsening in performance [44]. While the first effect could be due to a lack of sufficient diversity in the synthetic data, the latter is a consequence of unrepresentative synthetic data. Therefore, we hypothesize that performance will increase up to a certain ratio of synthetic to real data.

#### 4) Class-Specific Imbalance

There is no reason to believe that introducing an imbalance to one of the classes should outperform that introduced to any of the other classes. Therefore, we hypothesize that the results will be similar regardless of class-specific imbalance.

## VI Results

### A Performance of Proposed CGANs

We quantify the representativeness of the synthetic data via the MMD values in Table III, where a lower value implies that the synthetic data is more realistic. An average is taken over 10 seeds with each seed containing 30 (15) randomly sampled datapoints from the appropriate distributions of the HFM (CVD) dataset. Fewer samples are chosen for the CVD dataset due to the small sample size in the original dataset. We also propose the use of cMMD values in order to discern interclass differences. Such a granular approach facilitates the identification of potential causal relationships between network/hyperparameter changes and representativeness of synthetic data. This can ultimately guide researchers working with *conditional* GANs. When considering all classes, we can observe that MADGAN generates data that most resembles the true underlying distribution for both datasets. A closer look at the HFM cMMD values, however, indicates that CGAN+DS is able to produce the most realistic class 1 data. Conversely, DeLiGAN+DS appears to generate the least realistic synthetic data as observed by its relatively high cMMD and MMD values. We believe such a situation may arise due to the over-powering effect of the constraints placed on the DeLiGAN+DS network such as the diversity-sensitivity loss. In other words, the network could have placed greater emphasis on generating diverse classes compared to generating realistic classes. We also compare the real and synthetic data by visualizing them in a 2-dimensional t-SNE [58] subspace and calculating the pairwise L2 distance between them. More details can be found in Section-III of the Supplementary Material.

**Fig. 3:**
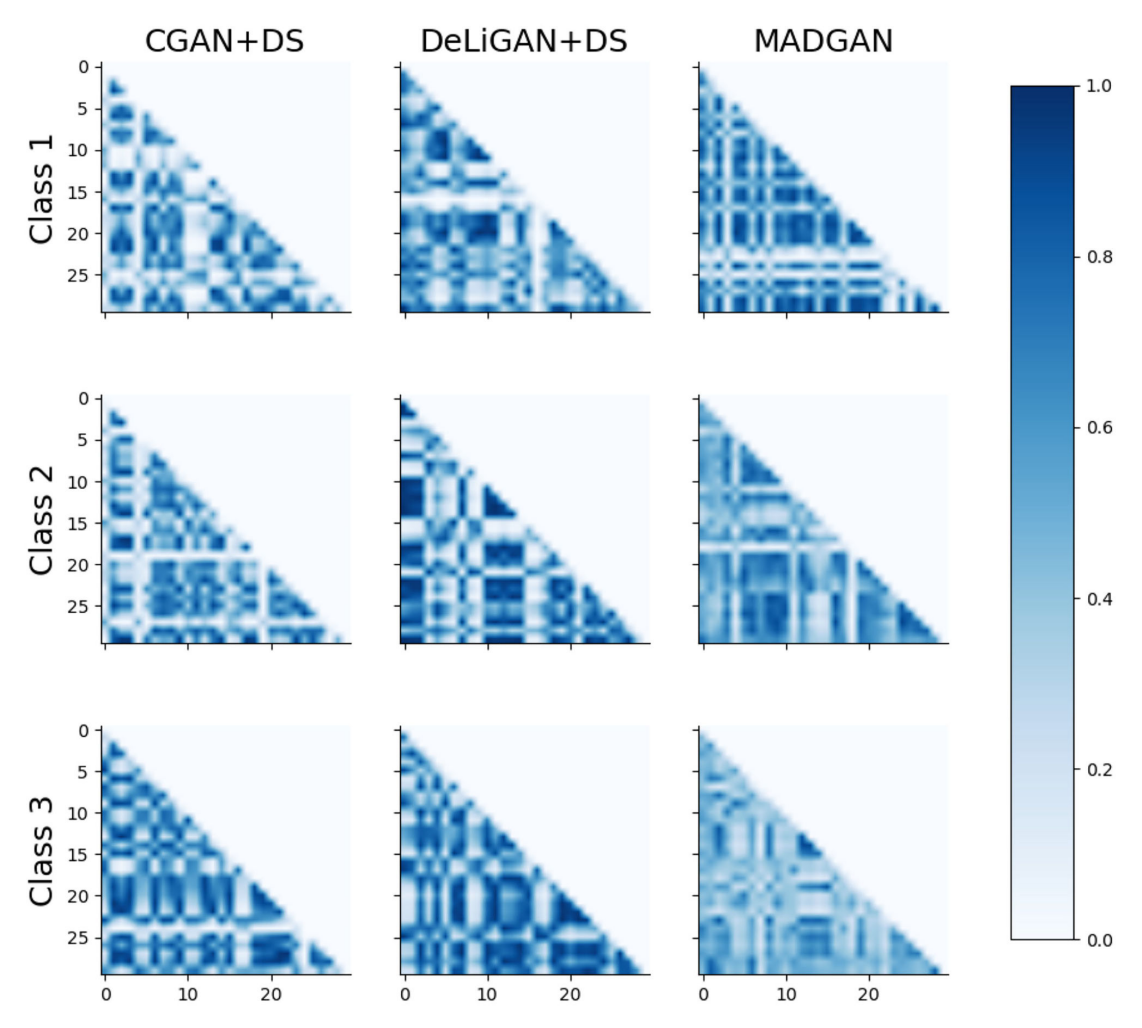
Lower triangular exponentiated quadratic kernel matrices representing the intraclass similarity of 30 randomly sampled synthetic datapoints generated by the three different CGANs (columns) for each of the three classes of HFM (rows). Results are shown for one seed.

In addition to representativeness of the synthetic data, we must ensure that the CGANs are not suffering from mode-collapse i.e. data generated from each class must be sufficiently diverse. This diversity is illustrated in Fig. 3 where the exponentiated quadratic kernel is applied to 30 randomly sampled synthetic datapoints from each class and model combination. For each such combination, the resulting symmetric matrix is truncated to only show its lower triangular region. Darker elements indicate synthetic datapoints that are quite similar to one another; a potential sign of class-specific mode-collapse. Conversely, lighter values indicate datapoints that are dissimilar from one another. Although this hints at the existence of intra-class diversity, it could also be a sign that the synthetic datapoint should not even belong to that class. This latter case would confuse classification models and negatively impact their performance. The intraclass similarity matrices belonging to the remaining datasets can be found in Section-IV of the Supplementary Material.

We calculate an intra-class similarity score in Table IV by taking the average of the off-diagonal elements of the 30 × 30 kernel matrices. Moreover, we mitigate the impact of a small sample size by averaging this across 10 different sets of 30 randomly sampled synthetic datapoints. Since we are aiming for high diversity, or equivalently low similarity, the lower the value the better. Based on this intuition, we can observe that CGAN+DS on the HFM dataset suffers the least from mode-collapse when generating data from class 1. The poorer diversity observed in class 3 implies that its generator became more focused on the conditional component of the input than on the random variable. It is worthwhile to note the correlation of the results in Table III and Table IV. We observe that the most diverse scenarios are the ones that correspond to the most representative synthetic data. Such a finding supports the notion that encouraging diversity can be advantageous.

### B Effect of Data Augmentation - Results

#### Augmentation Methods

**Fig. 4:**
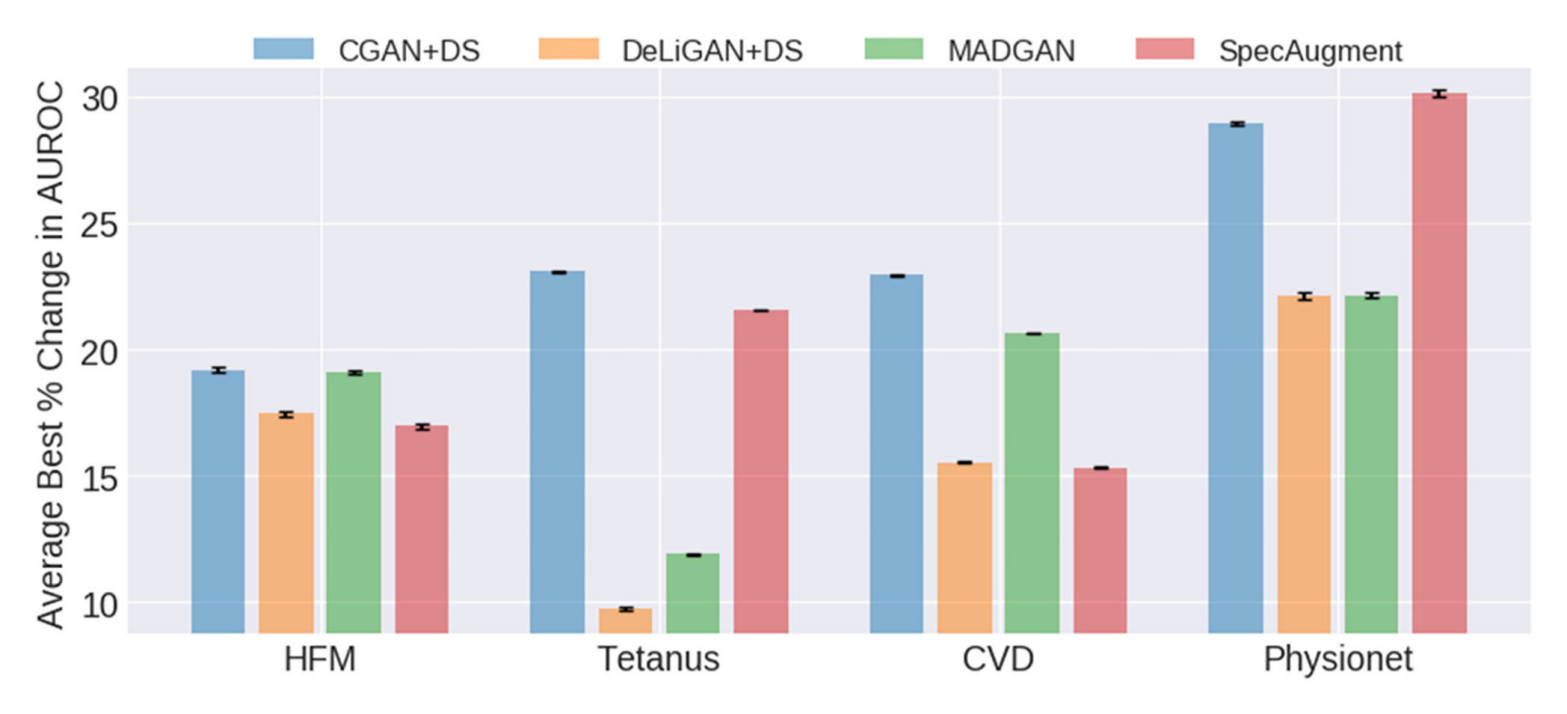
Average best percent change in AUROC as a function of the different augmentation methods used on each dataset. Error bars represent one standard error.

GAN-based data augmentation can improve classification performance relative to a balanced sub-sampled baseline by up to 29% as illustrated in Fig. 4. The absolute classification performance before and after augmentation can be found in Section-VII of the Supplementary Material.

Firstly, we observe that the ranking of the three GAN-based methods are consistent across the four datasets, with CGAN+DS outperforming the others (*p* < 0.05). Such consistency is promising and is indicative of the robustness of these models. We explain the relatively poorer behaviour of the remaining GANs by noting the potential limitations of artificially inducing interclass diversity when originally present to a minimal extent. Furthermore, for three of the four datasets (HFM, Tetanus, and CVD), our GAN-based data augmentation outperforms that of SpecAugment in a statistically significant manner (*p* < 0.05). On the Physionet dataset, the difference between the SpecAugment and CGAN+DS results are not statistically significant. We attribute the strong performance of the GANs to their ability to generate representative and sufficiently diverse synthetic data. When performance is relatively worse than SpecAugment, as in the case of Physionet, we attribute this to the high degree of noise present within the dataset and also to the inability of the GANs to generate realistic datapoints (see Section-II of Supplementary Material). Lastly, the CGAN+DS and MADGAN appear to produce more consistent outcomes across datasets. This increased reliability may be a positive trait among practitioners.

#### Training Set Imbalance

For all datasets except Physionet, we were somewhat surprised to observe that there is no significant difference in the results generated by balanced and unbalanced training sets. This could be explained by certain synthetic classes being less diverse and informative than others, a finding supported by the intraclass diversity plots. Therefore, more samples from only that class would be needed to improve performance.

#### Ratio of Synthetic to Real Data

After performing an ANOVA and a Kruskal-Wallis test, we observe that there is no significant difference between the results generated by a variety of synthetic to real data ratios. This implies that the utility of the synthetic data is limited, at least for the range of ratios chosen. The improvement in classification performance, however, indicates that only a small amount of synthetic data can have a strong positive impact. Such a finding was most prominent for the Physionet dataset.

#### Class Imbalance

On the HFM dataset, we observe that the classification improvement caused by introducing an imbalance in class 1 significantly outperforms (*p* < 0.05) that when imbalances are introduced in other classes. Anticipating such a potential outcome, based on work in [59], the CGANs were trained with a balanced dataset to avoid class favouritism. Nevertheless, this effect is still observed and can be partly explained by the relatively strong class 1 cMMD values relative to the others as seen in Table III. This phenomenon, however, is not observed with the other datasets. The figures associated with the aforementioned hypotheses can be found in Section-V of the Supplementary Material.

**Fig. 5:**
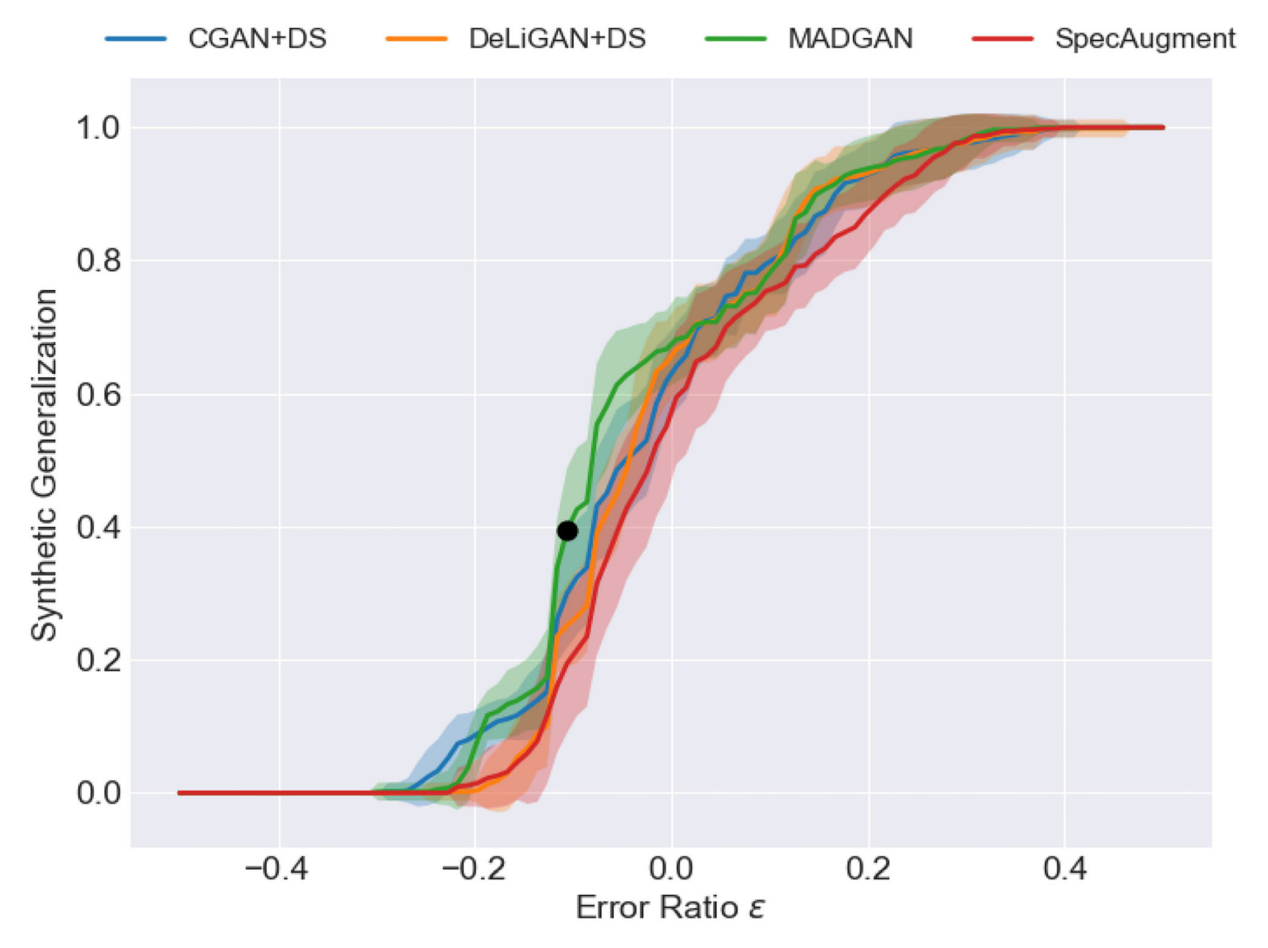
Synthetic generalization curve averaged across all 54 augmentation policies for each augmentation method when tested on the CVD dataset. Shaded area represents one standard deviation from the mean.

Data augmentation, although sometimes beneficial, can also be detrimental. To better understand the potential improvement *and* worsening of classification due to data augmentation, we illustrate our novel synthetic generalization curve in Fig. 5. Analagous to an ROC curve, the higher it is, the stronger the outcome. Moreover, increased mass when *ε* < 0 is indicative of classification improvement relative to the chosen baseline. For instance, the black dot indicates that when using MADGAN to augment the dataset, 40% of the classification models on average perform equivalent to or better than 1.10 of the baseline performance. We also observe that all methods are upper-bounded by the MADGAN method, indicating the latter’s superiority. The synthetic generalization curves for the remaining datasets can be found in Section-VI of the Supplementary Material.

Building on the analogy to the ROC, we introduce the AUSGC values in Table V. Given the range of values chosen for epsilon, the closer the AUSGC is to 1, the better the conditional GAN is in improving classification. Moreover, the smaller the standard deviation, the more consistent the conitional GAN is across the chosen augmentation policies. In other words, it is not producing highly varying behaviour. Ultimately, no statistical difference was found between the AUSGC values of the various augmentation methods. Nonetheless, we would like to emphasize that although we have used AUROC as the comparative performance metric in (9), this curve is inherently *metric agnostic*; i.e., one can use any performance metric. This allows researchers to choose their metric of interest based on the task at hand.

## VII Conclusion

Challenges posed by insufficient medical time-series data which are class-imbalanced can limit the potential of clinical decision support algorithms. To overcome such challenges, we modify and compare various conditional generative adversarial networks in their ability to synthesize pathological photoplethysmogram data. If researchers are solely aiming to generate the most realistic PPG data, then we recommend the DeLiGAN+DS and MADGAN methods in light of the relatively lower maximum mean discrepancy and L2 distance values. If, however, researchers are *also* aiming to boost the performance of their classification task, then we recommend CGAN+DS. Using this method, we show a statistically significant improvement of the AUROC by up to 29%.

For researchers working with time-series in low-data regimes, our proposed models offer them an opportunity to expand their dataset and improve their classification performance. Unfortunately, in pursuit of rules of thumb for augmentation policies, we were unable to find significant patterns. Future work would involve the evaluation of such augmentation methods on more complex neural networks and the *simultaneous* generation of different pathological medical time-series data. Also, the merger of generative modelling with self-supervised learning can be leveraged to obtain clinically acceptable classification performance.

## Supplementary Material

supp1-2979608

supp2-2979608

supp3-2979608

supp4-2979608

supp5-2979608

## Figures and Tables

**Table I:** Dataset-Specific Cross-Validation Summary

Dataset	nFolds	Input Dimensionality	Class Ratios
HFM - Vietnam	16	1980 x 50	1.35 : 1.04 : 1
Tetanus - Vietnam	2	5978 x 50	1 : 4.75 : 2.76
CVD - China [48]	10	219 x 50	8.25 : 1.7 : 1
Physionet [50]	7	2202 x 50	4.49 : 3.64 : 1

**Table II:** Network Architecture Common to All 3 CGAN Models

Network	Layer	Dimension	Activation
Generator	Input	50	-
	FC1	100	tanh
	FC2	200	tanh
	FC3	400	tanh
	FC4	500	tanh
	Conv1d	500	-
Discriminator	Input	500*^p^	-
	FC1	400	tanh
	FC2	200	tanh
	FC3	100	tanh
	FC4	50	tanh
	FC5a	1	-
	FC5b	3	-

p represents the packing degree introduced in [53]. FC and Conv1d represent Fully Connected and 1d Convolution operations, respectively.

**Table III:** Average Maximum Mean Discrepancy of Synthetic Data

Dataset	Class	CGAN+DS	DeLiGAN+DS	MADGAN
HFM	1	**0.84±0.089**	0.87±0.066	0.89±0.089
	2	0.85±0.085	0.97±0.087	**0.85±0.066**
	3	0.94±0.046	1.03±0.033	**0.85±0.034**
	All	0.87±0.048	0.90±0.032	**0.69±0.034**
Tetanus	1	0.50±0.040	0.52±0.029	**0.25±0.023**
	2	0.53±0.040	0.69±0.022	**0.36±0.023**
	3	0.50±0.038	0.69±0.041	**0.44±0.027**
	All	0.50±0.017	0.60±0.031	**0.26±0.011**
CVD [48]	1	0.88±0.082	1.05±0.089	**0.81**±**0.078**
	2	0.92±0.089	1.11±0.120	**0.89±0.100**
	3	**0.85±0.086**	0.87±0.067	0.91±0.099
	All	0.88±0.038	0.91±0.054	**0.66±0.034**
Physionet [50]	1	0.69±0.053	0.48±0.048	**0.40±0.050**
	2	0.76±0.043	**0.51±0.036**	0.51±0.040
	3	0.73±0.060	0.58±0.051	**0.47±0.070**
	All	0.72±0.031	0.49±0.019	**0.40±0.031**

**Table IV:** Average Intraclass Similarity of Synthetic Data

Dataset	Class	CGAN+DS	DeLiGAN+DS	MADGAN
HFM	1	**0.46±0.044**	0.49±0.039	0.51±0.034
	2	0.47±0.046	0.59±0.043	**0.47±0.024**
	3	0.55±0.051	0.64±0.025	**0.46±0.019**
Tetanus	1	0.44±0.025	0.38±0.019	**0.31±0.021**
	2	0.45±0.026	0.49±0.018	**0.42±0.022**
	3	0.44±0.020	0.50±0.021	**0.43±0.036**
CVD [48]	1	0.53±0.081	0.70±0.089	**0.47±0.070**
	2	0.51±0.084	0.70±0.099	**0.48±0.081**
	3	**0.43±0.044**	0.45±0.045	0.50±0.087
Physionet [50]	1	0.42±0.036	0.42±0.027	**0.34±0.027**
	2	0.42±0.033	0.47±0.025	**0.41±0.031**
	3	0.42±0.044	0.50±0.031	**0.42±0.024**

**Table V:** AUSGC Averaged Across All 54 Augmentation Policies

Dataset	CGAN+DS	DeLiGAN+DS	MADGAN	Spec Augment [31]
HFM	0.511±0.014	0.521±0.014	**0.525±0.018**	0.516±0.014
Tetanus	0.522±0.011	0.509±0.012	0.510±0.010	**0.540±0.010**
CVD [48]	0.521±0.014	0.512±0.015	**0.534±0.016**	0.490±0.021
Physionet [50]	0.521±0.013	0.517±0.012	0.500±0.012	**0.581 ±0.027**

## References

[1] Jeni LA, Cohn JF, De La Torre F (2013). Facing imbalanced data–recommendations for the use of performance metrics.

[2] Mazurowski MA, Habas PA, Zurada JM, Lo JY, Baker JA, Tourassi GD (2008). Training neural network classifiers for medical decision making: The effects of imbalanced datasets on classification performance. Neural Netw.

[3] Elgendi M, Liang Y, Ward R (2018). Toward generating more diagnostic features from photoplethysmogram waveforms. Diseases.

[4] Marcus G (2018). Deep learning: A critical appraisal.

[5] Wang J, Perez L (2017). The effectiveness of data augmentation in image classification using deep learning. Convolutional Neural Networks Vis Recognit.

[6] Russakovsky O (2015). Imagenet large scale visual recognition challenge. Int J Comput Vision.

[7] Ronneberger O, Fischer P, Brox T (2015). U-net: Convolutional networks for biomedical image segmentation.

[8] Frid-Adar M, Klang E, Amitai M, Goldberger J, Greenspan H (2018). Synthetic data augmentation using GAN for improved liver lesion classification.

[9] Um TT (2017). Data augmentation of wearable sensor data for parkinson’s disease monitoring using convolutional neural networks.

[10] Antoniou A, Storkey A, Edwards H (2017). Data augmentation generative adversarial networks.

[11] Mirza M, Osindero S (2014). Conditional generative adversarial nets.

[12] Gurumurthy S, Kiran Sarvadevabhatla R, Venkatesh Babu R (2017). Deligan: Generative adversarial networks for diverse and limited data.

[13] Ghosh A, Kulharia V, Namboodiri VP, Torr PH, Dokania PK (2018). Multi-agent diverse generative adversarial networks.

[14] Goodfellow IJ (2014). Generative adversarial networks.

[15] Salehinejad H, Colak E, Dowdell T, Barfett J, Valaee S (2019). Synthesizing chest x-ray pathology for training deep convolutional neural networks. IEEE Trans Med Imag.

[16] Yi X, Walia E, Babyn P (2019). Generative adversarial network in medical imaging: A review. Med Image Anal.

[17] Chen Y, Wang Y, Kirschen D, Zhang B (2018). Model-free renewable scenario generation using generative adversarial networks. IEEE Trans Power Syst.

[18] Wang F, Zhong S-H, Peng J, Jiang J, Liu Y, Schoeffmann K, Chalidabhongse TH, Ngo CW, Aramvith S, Ho Y-S, Gabbouj M, Elgammal A (2018). MultiMedia Modeling.

[19] Dong H-W, Hsiao W-Y, Yang L-C, Yang Y-H (2018). Musegan: Multi-track sequential generative adversarial networks for symbolic music generation and accompaniment.

[20] Gupta A, Johnson J, Fei-Fei L, Savarese S, Alahi A (2018). Social GAN: Socially acceptable trajectories with generative adversarial networks.

[21] Gregor Hartmann K, Tibor Schirrmeister R, Ball T (2018). EEG-GAN: Generative adversarial networks for electroencephalograhic (EEG) brain signals.

[22] Pascual D, Aminifar A, Atienza D, Ryvlin P, Wattenhofer R (2019). Synthetic epileptic brain activities using generative adversarial networks.

[23] Aznan NKN, Atapour-Abarghouei A, Bonner S, Connolly J, Moubayed NA, Breckon T (2019). Simulating brain signals: Creating synthetic EEG data via neural-based generative models for improved SSVEP classification.

[24] Severo D, Amaro F, Hruschka ER, Costa ASdM (2019). Ward2icu: A vital signs dataset of inpatients from the general ward.

[25] Brophy E, Wang Z, Ward TE (2019). Quick and easy time series generation with established image-based GANs.

[26] Zhang Q, Liu Y (2018). Improving brain computer interface performance by data augmentation with conditional deep convolutional generative adversarial networks.

[27] Esteban C, Hyland SL, Rätsch G (2017). Real-valued (Medical) time series generation with recurrent conditional GANs.

[28] Yang D, Hong S, Jang Y, Zhao T, Lee H (2019). Diversity-sensitive conditional generative adversarial networks.

[29] Krizhevsky A, Sutskever I, Hinton GE (2017). Imagenet classification with deep convolutional neural networks. Commun ACM.

[30] Wang Z, Qu Y, Tao J, Song Y (2019). Image-mediated data augmentation for low-resource human activity recognition.

[31] Park DS (2019). SpecAugment: A simple data augmentation method for automatic speech recognition.

[32] Guennec AL, Malinowski S, Tavenard R (2016). Data augmentation for time series classification using convolutional neural networks.

[33] Fawaz HI, Forestier G, Weber J, Idoumghar L, Muller P-A (2018). Data augmentation using synthetic data for time series classification with deep residual networks.

[34] Thickstun J, Harchaoui Z, Foster D, Kakade SM (2018). Invariances and data augmentation for supervised music transcription.

[35] McFee B, Humphrey EJ, Bello JP (2015). A software framework for musical data augmentation.

[36] Mauch M, Ewert S (2013). The audio degradation toolbox and its application to robustness evaluation.

[37] DeVries T, Taylor GW (2017). Dataset Augmentation in Feature Space.

[38] Oh J, Wang J, Wiens J (2018). Learning to exploit invariances in clinical time-series data using sequence transformer networks.

[39] Salimans T, Goodfellow I, Zaremba W, Cheung V, Radford A, Chen X (2016). Improved techniques for training GANs.

[40] Arjovsky M, Chintala S, Bottou L (2017). Wasserstein generative adversarial networks.

[41] Thanh-Tung H, Tran T, Venkatesh S (2019). Improving generalization and stability of generative adversarial networks.

[42] Borji A (2019). Pros and cons of GAN evaluation measures. Comput Vision Image Understanding.

[43] Gretton A, Borgwardt KM, Rasch MJ, Schölkopf B, Smola A (2012). A kernel two-sample test. J Mach Learn Res.

[44] Tran T, Pham T, Carneiro G, Palmer L, Reid I (2017). A bayesian data augmentation approach for learning deep models.

[45] Khanh TH (2012). Enterovirus 71-associated hand, foot, and mouth disease, Southern Vietnam, 2011. Emerg Infectious Diseases.

[46] Hoang MTV (2019). Clinical and aetiological study of hand, foot and mouth disease in Southern Vietnam, 2013–2015: Inpatients and outpatients. Int J Infectious Diseases.

[47] Ablett J (1967). Analysis and main experience in 82 patients treated in leeds tetanus unit.

[48] Liang Y, Chen Z, Liu G, Elgendi M (2018). A new, short-recorded photoplethysmogram dataset for blood pressure monitoring in china. Scientific Data.

[49] Liang Y, Liu G, Chen Z, Elgendi M (2018). PPG-BP database.

[50] Goldberger AL (2000). Physiobank, physiotoolkit, and physionet: Components of a new research resource for complex physiologic signals. Circulation.

[51] T. F. of the European Society of Cardiology, the North American Society of Pacing, and Electrophysiology (1996). Heart rate variability: Standards of measurement, physiological interpretation and clinical use. Circulation.

[52] Bellman RE (1961). Adaptive Control Processes: A Guided Tour.

[53] Lin Z, Khetan A, Fanti G, Oh S (2018). PacGAN: The power of two samples in generative adversarial networks.

[54] Donahue C, McAuley J, Puckette M (2018). Adversarial audio synthesis.

[55] Charlton PH (2017). Extraction of respiratory signals from the electro-cardiogram and photoplethysmogram: Technical and physiological determinants. Physiological Meas.

[56] van der Walt CM, Barnard E (2006). Data characteristics that determine classifier performance. SAIEE Africa Research J.

[57] Pinto N, Cox DD, DiCarlo JJ (2008). Why is real-world visual object recognition hard?. PLoS Comput Biol.

[58] Maaten Lvd, Hinton G (2008). Visualizing data using T-SNE. J Mach Learn Res.

[59] Mariani G, Scheidegger F, Istrate R, Bekas C, Malossi C (2018). BaGAN: Data augmentation with balancing GAN. CoRR.

