# Supplementary material for: PlethAugment: GAN-Based PPG Augmentation for Medical Diagnosis in Low-Resource Settings
Source: IEEE J Biomed Health Inform. Author manuscript; Available in PMC 2025 Sep 29. (PMC7618182; doi:10.1109/JBHI.2020.2979608)
Supplement: supp4-2979608 [file EMS208285-supplement-supp4_2979608.pdf]

# Supplementary: PlethAugment: GAN-Based PPG Augmentation for Medical Diagnosis in Low-Resource Settings

Dani Kiyasseh, Girmaw Abebe Tadesse, Le Nguyen Thanh Nhan, Le Van Tan, Louise Thwaites, Tingting Zhu*, and David Clifton*

## I. Effect of Diversity Sensitivity Term on GAN Evaluation Metrics

In this section, we illustrate the effect of the diversity sensitivity term on the visual appearance of the PPG signal and its embedding in a 2-dimensional PCA subspace. We also quantify the impact on the average maximum mean discrepancy (MMD) and the intraclass similarity. In the former metric, a lower value is indicative of more realistic datapoints. In the latter, a lower value is indicative of reduced mode-collapse.

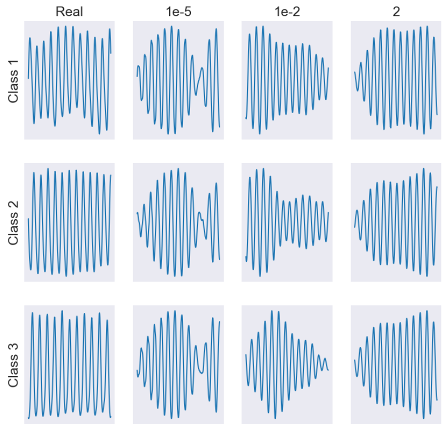


Fig. 1: Randomly sampled class-specific real (HFM) and synthetic PPG data generated by models trained with different degrees of diversity sensitivity. Samples are 5s in duration.

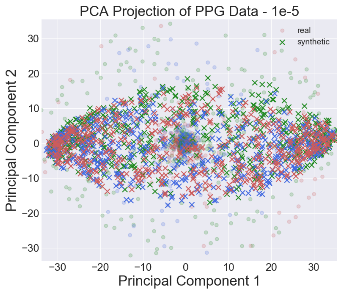


(a) 1e-5

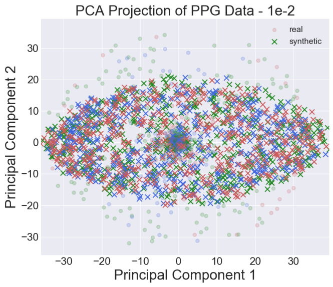


(b) 1e-2

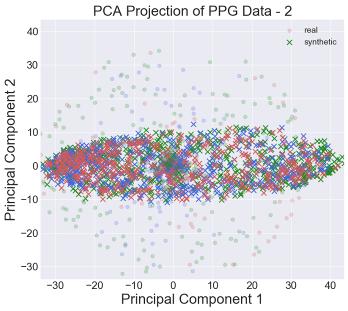


(c) 2

Fig. 2: PCA projection of the real and synthetic datapoints generated by CGAN+DS at the end of training with varying degrees of diversity sensitivity $\lambda_{div} = 1e-2, 1e-5, 2$. The 3 different classes are represented by the 3 colours. Videos of the PCA projection of samples generated during training can be found in the multimedia attachments.

TABLE I: Average maximum mean discrepancy of synthetic data from CGAN+DS when trained with different degrees of diversity sensitivity.

| Dataset | Class | $\lambda_{div}$ 1e-5 | 1e-2 | 2 |
|---|---|---|---|---|
| HFM | 1 | 0.71±0.054 | 0.72±0.042 | **0.70±0.063** |
| | 2 | **0.73±0.056** | 0.74±0.068 | 0.73±0.071 |
| | 3 | 0.70±0.065 | **0.68±0.055** | 0.71±0.069 |
| | All | 0.71±0.023 | 0.71±0.023 | 0.71±0.044 |

TABLE II: Average intraclass similarity of synthetic data from CGAN+DS trained with different degrees of diversity sensitivity.

| Dataset | Class | $\lambda_{div}$ 1e-5 | 1e-2 | 2 |
|---|---|---|---|---|
| HFM | 1 | 0.35±0.045 | 0.35±0.052 | **0.33±0.057** |
| | 2 | **0.36±0.031** | 0.37±0.047 | 0.37±0.059 |
| | 3 | 0.34±0.024 | **0.33±0.039** | 0.36±0.063 |

## II. Real and Fake PPG Data Comparison

This section contains the real and fake PPG data for the remaining datasets.

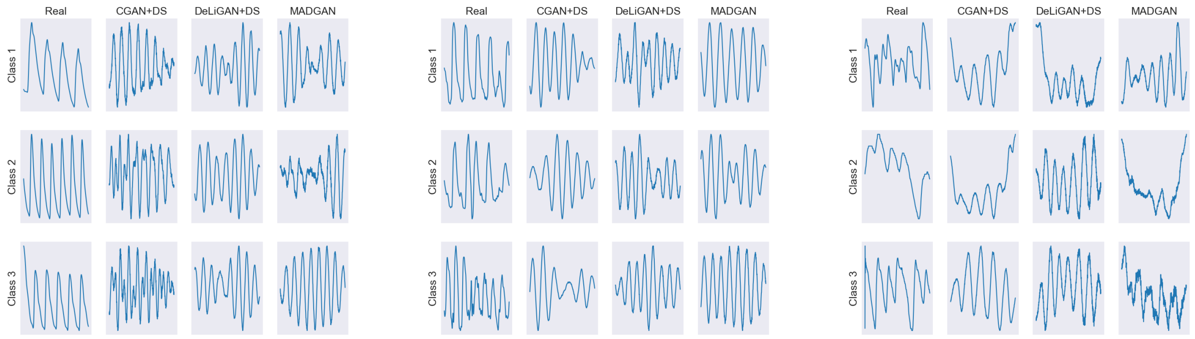


(a) Tetanus (b) CVD (c) Physionet

Fig. 3: Randomly sampled class-specific real and synthetic PPG data generated by each of the CGAN models. Samples are 5 s in duration. Note the ability of the CGANs to capture respiratory sinus arrythmia-induced amplitude modulation.

## III. Comparison of Real Data to Synthetic Data in t-SNE Subspace

In this section, we perform non-linear dimensionality reduction on the real and synthetic PPG signals using t-distributed Stochastic Neighbour Embedding (t-SNE) [1]. The results are shown for each of the four datasets (HFM, Tetanus, CVD, and Physionet) and each of the GANs implemented (CGAN+DS, DeLIGAN+DS, and MADGAN). The three colors represent the three different classes for each dataset. Once the data is in an embedded space, we normalize the features and calculate the average pairwise L2 distance between each real and synthetic datapoint that belong to the same class.

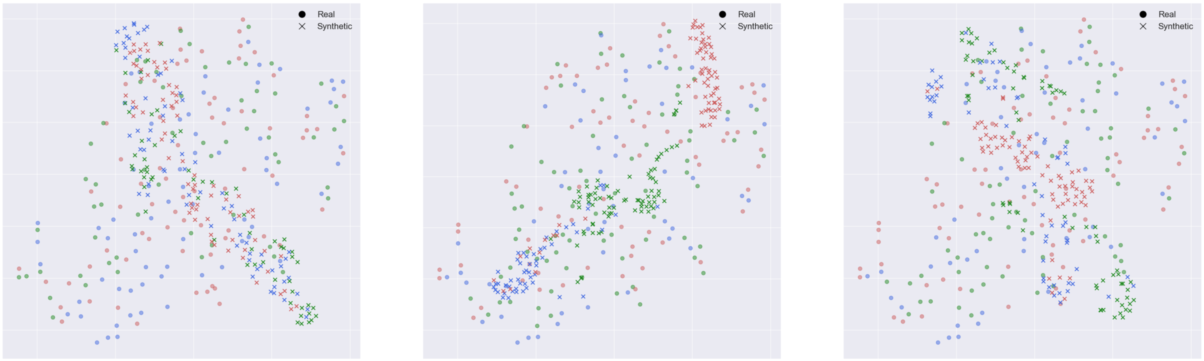


(a) CGAN+DS (b) DeLiGAN+DS (c) MADGAN

Fig. 4: t-SNE embedding of real and synthetic data from HFM dataset

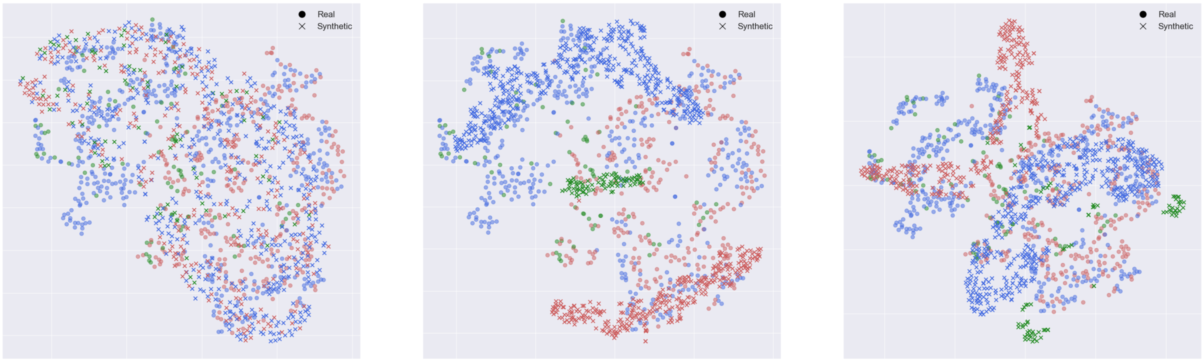


(a) CGAN+DS (b) DeLiGAN+DS (c) MADGAN

Fig. 5: t-SNE embedding of real and synthetic data from Tetanus dataset

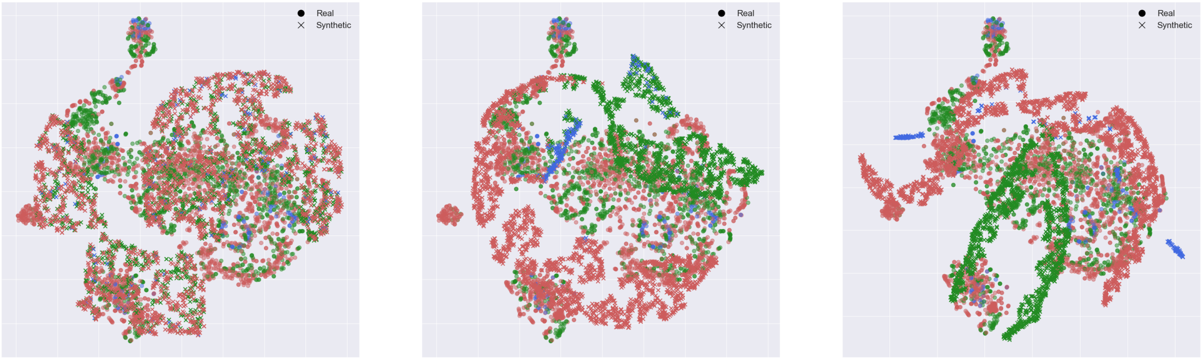


(a) CGAN+DS (b) DeLiGAN+DS (c) MADGAN

Fig. 6: t-SNE embedding of real and synthetic data from Physionet dataset

TABLE III: Average pairwise Euclidean distance between normalized t-SNE embedding of real and synthetic data

| Dataset | Class | CGAN+DS | DeLiGAN+DS | MADGAN |
|---|---|---|---|---|
| HFM | 1 | 1.95 | **1.37** | 2.04 |
| | 2 | **1.79** | 2.17 | 1.93 |
| | 3 | 1.77 | 1.94 | **1.56** |
| | All | 1.84 | **1.83** | 1.84 |
| Tetanus | 1 | 1.48 | **1.16** | 2.27 |
| | 2 | 1.89 | **1.70** | 1.86 |
| | 3 | 1.78 | **1.52** | 2.19 |
| | All | 1.72 | **1.46** | 2.11 |
| CVD [2] | 1 | **1.79** | 1.83 | 1.80 |
| | 2 | 1.93 | **1.48** | 1.60 |
| | 3 | 1.88 | **1.71** | 2.02 |
| | All | 1.87 | **1.67** | 1.81 |
| Physionet [3] | 1 | 1.74 | 1.70 | **1.67** |
| | 2 | 1.97 | **1.80** | 2.12 |
| | 3 | **1.84** | 1.85 | 1.91 |
| | All | 1.85 | **1.78** | 1.90 |

In the t-SNE plots, the presence of relatively dense regions that contain synthetic datapoints (as in Fig. 4b) could be indicative of mode-collapse. This is a common challenge posed by GANs where trained generators produce outputs that are similar to one another and that do not span the entire distribution of the real data. When evaluating the Euclidean distance between class-specific datapoints in Table III, we observe that, on average, DeLiGAN+DS produces synthetic datapoints that are most similar to their real counterparts. These findings in addition to those in Table III in the main mauscript suggest that, if the sole aim of researchers is to generate realistic class-specific PPG data, then DeLiGAN+DS and MADGAN are the preferred models. If, however, one is aiming to generate relatively realistic PPG data that *also* benefits the classification performance of models the most, then we recommend using CGAN+DS. This recommendation is based on the results in Fig. 4 in the main manuscript that illustrate CGAN+DS's superiority in terms of average performance relative to the other methods.

## IV. Intraclass Similarity Matrices

This section contains the intraclass similarity kernel matrices for the remaining datasets.

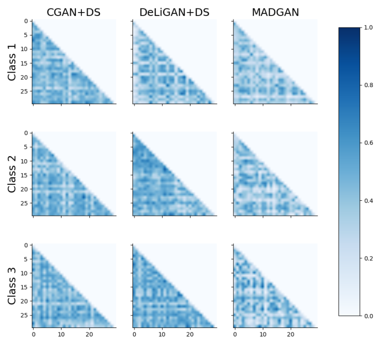


(a) Tetanus

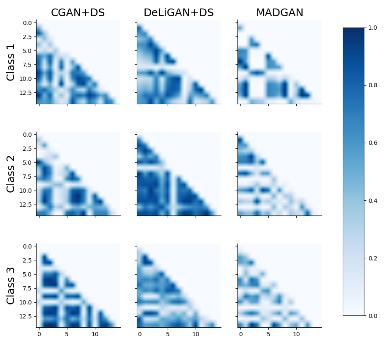


(b) CVD

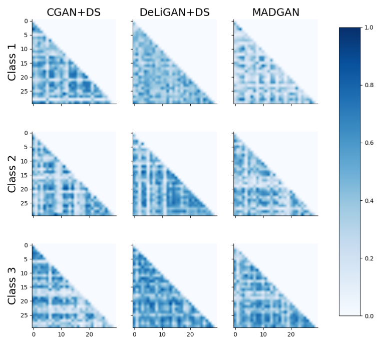


(c) Physionet

Fig. 7: Lower triangular exponentiated quadratic kernel matrices representing the intraclass similarity of 30 randomly sampled synthetic datapoints generated by the three different CGANs (columns) for each of the three classes. Results are shown for one seed.

## V. Plots Corresponding to Hypotheses

This section contains the plots associated with the hypotheses posed in the manuscript.

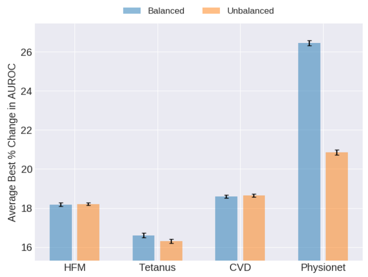


(a) Balance vs. Imbalance

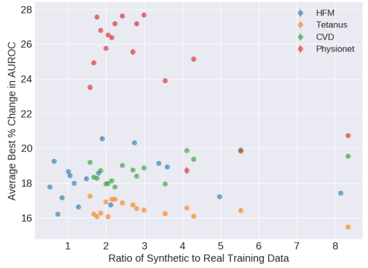


(b) Ratio of Synthetic to Real

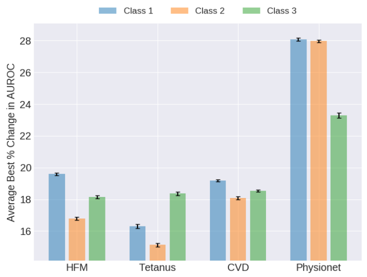


(c) Class Imbalance

Fig. 8: Plots associated with the hypotheses posed in the manuscript. The error bars represent one standard deviation from the mean.

## VI. Synthetic Generalization Curves

This section contains the synthetic generalization curves for the remaining datasets.

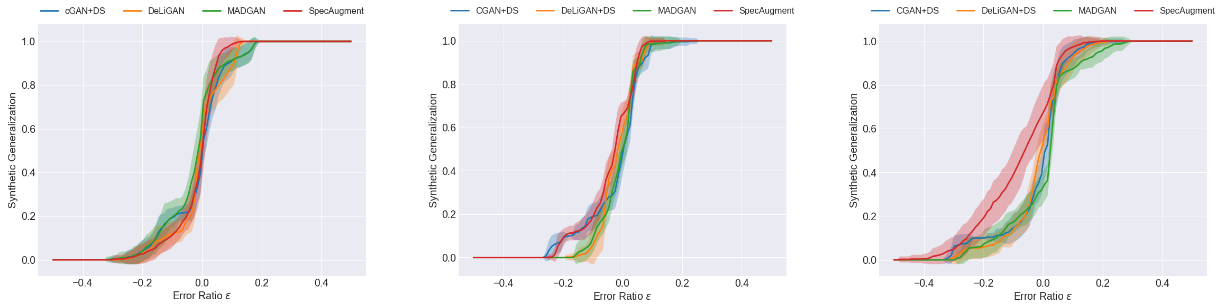


(a) HFM (b) Tetanus (c) Physionet

Fig. 9: Synthetic generalization curve averaged across all 54 augmentation policies for each augmentation method when tested on the target dataset. Shaded area represents one standard deviation from the mean.

## References


[1] L. v. d. Maaten and G. Hinton, "Visualizing data using t-sne," *Journal of machine learning research*, vol. 9, no. Nov, pp. 2579–2605, 2008.

[2] Y. Liang, Z. Chen, G. Liu, and M. Elgendi, "A new, short-recorded photoplethysmogram dataset for blood pressure monitoring in china," *Scientific data*, vol. 5, p. 180020, 2018.

[3] A. L. Goldberger, L. A. Amaral, L. Glass, J. M. Hausdorff, P. C. Ivanov, R. G. Mark, J. E. Mietus, G. B. Moody, C.-K. Peng, and H. E. Stanley, "Physiobank, physiotoolkit, and physionet: components of a new research resource for complex physiologic signals," *Circulation*, vol. 101, no. 23, pp. e215–e220, 2000.
